# Grain Yield and Quality of Foxtail Millet (*Setaria italica* L.) in Response to Tribenuron-Methyl

**DOI:** 10.1371/journal.pone.0142557

**Published:** 2015-11-13

**Authors:** Na Ning, Xiangyang Yuan, Shuqi Dong, Yinyuan Wen, Zhenpan Gao, Meijun Guo, Pingyi Guo

**Affiliations:** Laboratory of Crop Chemical Regulation and Chemical Weed Control, College of Agronomy, Shanxi Agricultural University, Taigu, Shanxi, the People's Republic of China; National Institute of Plant Genome Research, INDIA

## Abstract

Foxtail millet (*Setaria italica* L.) is cultivated around the world for human and animal consumption. There is no suitable herbicide available for weed control in foxtail millet fields during the post-emergence stage. In this study, we investigated the effect and safety of the post-emergence herbicide tribenuron-methyl (TBM) on foxtail millet in terms of grain yield and quality using a split-plot field design. Field experiments were conducted using two varieties in 2013 and 2014, i.e., high-yielding hybrid Zhangzagu 10 and high-quality conventional Jingu 21. TBM treatments at 11.25 to 90 g ai ha^−1^ reduced root and shoot biomass and grain yield to varying degrees. In each of the two years, grain yield declined by 50.2% in Zhangzagu 10 with a herbicide dosage of 45 g ai ha^−1^ and by 45.2% in Jingu 21 with a herbicide dosage of 22.5 g ai ha^−1^ (recommended dosage). Yield reduction was due to lower grains per panicle, 1000-grain weight, panicle length, and panicle diameter. Grain yield was positively correlated with grains per panicle and 1000-grain weight, but not with panicles ha^−1^. With respect to grain protein content at 22.5 g ai ha^−1^, Zhangzagu 10 was similar to the control, whereas Jingu 21 was markedly lower. An increase in TBM dosage led to a decrease in grain Mn, Cu, Fe, and Zn concentrations. In conclusion, the recommended dosage of TBM was relatively safe for Zhangzagu 10, but not for Jingu 21. Additionally, the hybrid variety Zhangzagu 10 had a greater tolerance to TBM than the conventional variety Jingu 21.

## Introduction

Foxtail millet (*Setaria italica* L.), a crop rich in nutrients, originated in China [1, 2]. Presently, foxtail millet is extensively cultivated as a food and fodder crop throughout Eurasia and the Far East [3]. Meanwhile, foxtail millet is being promoted as a model crop for cereal crops [4–8]. However, the yield and quality of crops are seriously affected by weed growth [9]. The competitions from weeds have been reported to reduce foxtail millet grain yield by as much as 55.56% [10]. Manual control with intensive labour is the main method to control weeds for the crop. Compared with conventional manual control of weeds, chemical control is less labor-intensive and more efficient. However, there is no suitable herbicide used for weed control in foxtail millet fields during the post-emergence stage.

Tribenuron-methyl (TBM) is a highly selective post-emergence sulfonylurea herbicide developed by the DuPont Company, USA. TBM belongs to the acetyl lactic acid synthase inhibitor, and can effectively control the vast majority of annual broadleaf weeds [11, 12]. There are significant differences in the sensitivity of wheat varieties to TBM [13]. Wheat varieties with high activity of glutathione S-transferase are more tolerant to TBM [14, 15]. At three-leaf stage of weeds, the recommended dosage of 75% TBM treatment shows better effect in broadleaf weed control and is relatively safe to hulless oat [16]. Rational application of TBM effectively inhibits weed growth, increases light penetration rate in fields, and enhances the yield of wheat; otherwise, it can seriously inhibit the yield and grain quality of wheat [17]. However, there is little evidence whether TBM is safe to foxtail millet crops or not.

Moreover, studies on chemical weed control in foxtail millet fields are rare. Application of 1800 g ha^−1^ of 44% monosulfuron plus propazine Wettable Powder (WP) at the pre-emergence stage can control the vast majority of broadleaf weeds and certain gramineae weeds in foxtail millet fields [18]. Sethoxydim is a safe post-emergence herbicide for gramineae weeds control in Zhangzagu 5, which has a sethoxydim resistant gene [19]. Additionally, with increasing monosulfuron concentrations, grain content of fat decreases while starch and amino acid contents increase in foxtail millet [20].

Grain yield and quality determine much of the value of cereal crops to the producers [21, 22]. Weeds can be controlled using herbicides [23], but certain crop damages are likely as the yield and quality affected by herbicides [24, 25]. Therefore, there is a need to study the effect of herbicides on crop grain yield and quality. Presently, there is little knowledge about the effect of the post-emergence herbicide TBM on the grain yield and quality of foxtail millet.

To address the above issue, the present study was carried out to investigate the effect and safety of the post-emergence herbicide TBM on the performance, yield, and grain quality of Zhangzagu 10 and Jingu 21. The variety Jingu 21, one of the main varieties of foxtail millet in China, which has been widely grown in the major foxtail millet production regions of northern China for 30 years, is a high-quality conventional variety. The variety Zhangzagu 10 is a new hybrid variety, whose one parent plant was obtained from improved Jingu 21, which showed enormous promotion potential in the world. The results will have certain guiding significance for the production and promotion of Zhangzagu 10.

## Materials and Methods

### Experimental location

Field study was conducted at the farm of Shanxi Agricultural University (37°42′ N and 112°58′ E), China, from May to October in 2013 and 2014. The study site has a temperate continental climate. The soil was a calcareous cinnamon soil. The main characteristics of the soil in the field prior to any treatment application are shown in Table 1. The annual on-site precipitation was approximately 400 mm, and the precipitation was higher in 2014 compared with 2013 during filling stages (August through September).

**Table 1:** Soil characteristics of the experimental fields.

Property	2013	2014
pH (H_2_O)	8.18	8.05
Organic matter (g kg^−1^)	19.11	16.31
Total N (g kg^−1^)	0.92	0.88
Available P (mg kg^−1^)	14.46	15.21
Available K (mg kg^−1^)	121.03	108.21
Available Mn (mg kg^−1^)	15.13	19.01
Available Cu (mg kg^−1^)	1.51	1.39
Available Fe (mg kg^−1^)	6.62	7.09
Available Zn (mg kg^−1^)	0.98	1.24

### Experimental design

Foxtail millet varieties used in the experiment were Zhangzagu 10 and Jingu 21, which are grown widely in Northern China. Zhangzagu 10 was obtained from the Zhangjiakou Academy of Agricultural Sciences of Hebei Province, China. Jingu 21 was supplied by the Institute of Economic Crops, Shanxi Academy of Agricultural Sciences, China. TBM (10%, WP) was provided by Shangbeng Lvye Chemical Co., Ltd., Shandong province, China.

The experiment was conducted as a split-plot with three replications. The main plot treatments were the two foxtail millet varieties. The subplot treatments were different dosages of TBM. Five test dosages (0, 11.25, 22.5, 45 and 90 g ai ha^−1^) of TBM were applied using laboratory pot-sprayers, previously calibrated to deliver 450 L ha^−1^, on foxtail millet seedlings at the five-leaf stage (most weeds at three-leaf stage). The recommended dosage for field application by the manufacturer was approximately 22.5 g ai ha^−1^. The plots were 6 m long by 3 m wide with row spacing every 0.315 m. The approximate density was 180,000 Zhangzagu 10 plants ha^−1^ and 300,000 Jingu 21 plants ha^−1^ (the field density recommended by the marketing company). Foxtail millet was planted on May 14^th^ 2013, and May 21^st^ 2014. The plots were weeded with hoes five times per year after a 2-week period following TBM application.

### Sampling

At maturity, tillers were counted. Samples of 30 panicles per plot (excluding the ones on the borders) were randomly collected for measurements of yield components. Each sample was threshed using panicle thresher and the grains were counted by grain counter and weighed. Samples for grain yield (adjusted to a moisture content of 14%) determination were obtained by harvesting the central 5-m^2^ areas of each plot to avoid border effects. The foxtail millet samples were dehulled twice using a rice huller (JLGJ4.5, Zhejiang Taizhou Food Instrument Factory, China). The dehulled samples were dried at 60°C for 48 h, ground mechanically (DFY-500A, Bilon, Shanghai, China), passed through a 0.5-mm sieve, and stored in a desiccator before chemical analyses.

### Chemical analysis

Soil pH was determined with a soil-to-water ratio of 1:2.5. Soil organic matter, total N, available P and K were quantified by potassium dichromate titration method, alkaline hydrolysis diffusion method, molybdenum antimony colorimetric method, flame photometry method, respectively [26]. Soil Fe, Mn, Cu and Zn concentrations were measured by inductively coupled plasma atomic emission spectrometry (5300DV, PE Company, USA).

Finely ground grain of foxtail millet (500 mg) was acid-digested with 5 mL of 70% superior-grade pure nitric acid and 2 mL of 30% hydrogen peroxide. Digested samples were diluted to 25 mL with ultrapure water (18.2 MΩ cm). Grain Mn, Cu, Fe and Zn concentrations were analysed by inductively coupled plasma atomic emission spectrometry [27] (5300DV, PE Company, USA). Grain protein concentration was measured by Kjeldahl using a 6.25 nitrogen-to-protein conversion factor.

### Statistical analysis

Statistics Analysis System 8.0 was used in statistical analysis of the data. Duncan’s test was used to determine the significant differences among the treatments. Simple correlation coefficients were calculated based on treatment means. We used *P* = 0.05 as the statistical significance threshold.

## Results

### Effect of TBM on foxtail millet biomass

**Fig 1:**
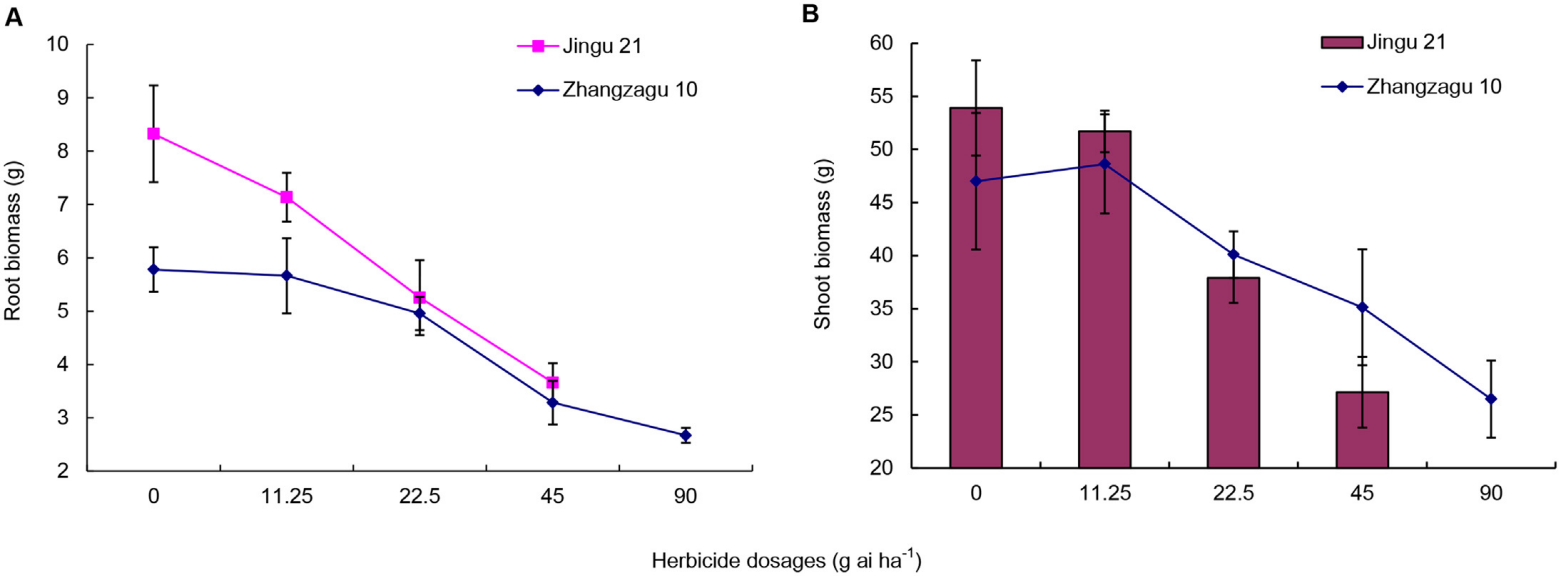
Effect of tribenuron-methyl (TBM) on foxtail millet biomass in 2013. Values represent means; vertical bars represent the standard deviation of three separate experiments. The abscissa in the figure represents the dosage of TBM expressed as g ai ha^−1^. Dead Jingu 21 plants were observed at 90 g ai ha^−1^ TBM.

**Fig 2:**
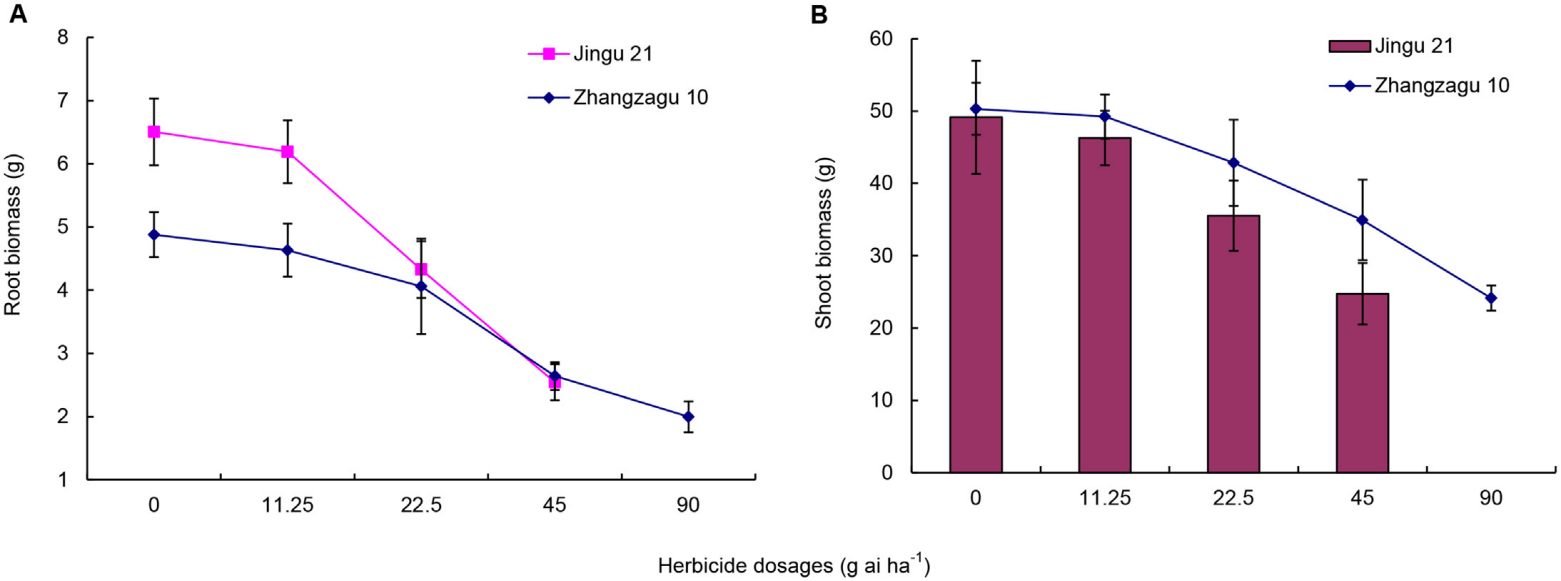
Effect of tribenuron-methyl (TBM) on foxtail millet biomass in 2014. Values represent means; vertical bars represent the standard deviation of three separate experiments. The abscissa in the figure represents the dosage of TBM expressed as g ai ha^−1^. Dead Jingu 21 plants were observed at 90 g ai ha^−1^ TBM.

TBM reduced root and shoot biomass of foxtail millet (Figs 1 and 2). In Zhangzagu 10, the reduction in root and shoot biomass (44.6% and 27.9%, respectively) was significant at 45 g ai ha^−1^ compared with the control; however, there was no significant difference between 11.25 and 22.5 g ai ha^−1^. In Jingu 21, the reduction in root and shoot biomass was significant at 22.5 g ai ha^−1^ (35.2% and 28.7%, respectively), but not at 11.25 g ai ha^−1^.

### Effect of TBM on yield and yield characteristics of foxtail millet

Yield and related characteristics of foxtail millet varied between years and varieties (Tables 2 and 3). Increasing dosages of TBM negatively affected yield in both years; however, the effects were more significant in Jingu 21 compared with Zhangzagu 10. The grain yield of Zhangzagu 10 was slightly lower than the control at 11.25 and 22.5 g ai ha^−1^ and significantly lower (50.2%) at 45 g ai ha^−1^. The grain yield of Jingu 21 significantly decreased (45.2%) at 22.5 g ai ha^−1^. Dead Jingu 21 plants were obtained at 90 g ai ha^−1^.

**Table 2:** Effects of tribenuron-methyl (TBM) on panicle length, panicle diameter, panicles ha^−1^, grains per panicle, 1000-grain weight and yield of foxtail millet Zhangzagu 10 in 2013 and 2014.

TBM (g ai ha^-1^)	Panicle length (cm)	Panicle diameter (mm)	Panicles number (×10^4^ ha^−1^)	Grains per panicle	1000-grain weight (g)	Yield (kg ha^−1^)
Zhangzagu 10 (2013)						
0	28.50±0.8a	34.25±1.0a	49±1.5a	7815±94.4a	3.32±0.09a	6105.27±183.8a
11.25	27.73±0.9a	32.61±1.1a	48±2.0a	7709±71.3a	3.25±0.2a	5954.43±137.3a
22.5	26.20±1.9a	30.34±0.6b	52±3.5a	7649±135.1a	3.19±0.2a	5759.85±241.8a
45	22.47±0.7b	25.23±0.6c	49±1.0a	5512±149.1b	2.80±0.2b	3004.51±393.0b
90	16.67±1.4c	20.08±1.4d	50±3.0a	4025±113.6c	2.90±0.1b	2220.29±231.3c
Zhangzagu 10 (2014)						
0	29.20±1.0a	35.17±2.0a	50±2.0a	8136±117.4a	3.26±0.04a	5887.73±283.8a
11.25	28.57±1.0a	33.50±1.2ab	47±2.5a	7955±79.7a	3.20±0.09a	5746.95±106.9a
22.5	26.80±1.6a	31.40±0.8b	46±4.0a	7847±63.5a	3.17±0.1a	5627.05±193.8a
45	22.17±1.5b	23.40±1.0c	47±2.5a	6034±176.0b	3.08±0.1ab	2966.82±171.6b
90	18.77±1.2c	19.23±1.1d	51±2.5a	4080±269.6c	2.92±0.1b	2526.79±136.5c

Values are expressed as mean ± SD (*n* = 3). Different letters in the same column represent significantly different at *P* = 0.05 level by Duncan’s new multiple range test.

**Table 3:** Effects of tribenuron-methyl (TBM) on panicle length, panicle diameter, panicles ha^−1^, grains per panicle, 1000-grain weight and yield of foxtail millet Jingu 21 in 2013 and 2014.

TBM (g ai ha^−1^)	Panicle length (cm)	Panicle diameter (mm)	Panicles number (×10^4^ ha^−1^)	Grains per panicle	1000-grain weight (g)	Yield (kg ha^−1^)
Jingu 21 (2013)						
0	25.73±0.8a	33.20±2.5a	43±3.5a	6607±347.6a	3.28±0.03a	4300.00±210.0a
11.25	23.67±0.5b	32.75±1.9a	38±1.5a	6207±163.7a	3.27±0.01a	3903.53±130.1b
22.5	20.53±0.6c	25.30±1.8b	42±3.5a	4937±72.9b	3.19±0.03b	2313.45±134.3c
45	15.80±1.5d	19.58±2.9c	38±2.7a	3595±204.5c	3.16±0.03b	1600.08±141.1d
90	−	−	−	−	−	−
Jingu 21 (2014)						
0	26.93±1.5a	34.68±1.8a	42±2.1a	7083±148.4a	3.15±0.06a	4076.43±232.9a
11.25	24.37±1.0a	31.40±1.3b	40±3.0a	6854±129.5a	3.09±0.1a	3887.77±138.5a
22.5	18.10±1.6b	24.53±1.4c	43±3.1a	5061±112.5b	2.80±0.2b	2277.70±223.7b
45	13.73±1.4c	20.81±1.0d	39±3.5a	3433±95.2c	2.51±0.1c	1481.33±196.2c
90	−	−	−	−	−	−

Values are expressed as mean ± SD (*n* = 3). Different letters in the same column indicate significantly different at *P* = 0.05 level by Duncan’s new multiple range test. “−” represents dead plants.

Increasing dosages of TBM negatively affected most of the yield characteristics tested (Tables 2 and 3). Zhangzagu 10 treated with dosages ≥45 g ai ha^−1^ showed significant differences from the control in terms of panicle length, panicle diameter, grains per panicle, and 1000-grain weight. On the other hand, panicles ha^−1^ was not affected by TBM treatments. In Jingu 21, TBM significantly decreased panicle length, panicle diameter, grain per panicle, and 1000-grain weight at dosages ≥11.25 g ai ha^−1^.

The relationship between yield and yield characteristics (panicle length, panicle diameter, panicles ha^−1^, grains per panicle, and 1000-grain weight) was similar between the two varieties (Table 4). In Zhangzagu 10 and Jingu 21, there was a significant positive correlation between yield and panicle length, panicle diameter, grains per panicle, or 1000-grain weight; however, no significant correlation was obtained between yield and panicles ha^−1^.

**Table 4:** Correlation coefficient between yield and panicle length, panicle diameter, panicles ha^−1^, grains per panicle, and 1000-grain weight of foxtail millet Zhangzagu 10 and Jingu 21.

Parameter	*x*_1_	*x*_2_	*x*_3_	*x*_4_	*x*_5_	*x*_6_
Zhangzagu 10						
*x*_1_	1.00					
*x*_2_	0.99**	1.00				
*x*_3_	-0.27	-0.29	1.00			
*x*_4_	0.98**	0.98**	-0.12	1.00		
*x*_5_	0.83*	0.89*	-0.13	0.88*	1.00	
*x*_6_	0.95**	0.97**	-0.11	0.99**	0.94**	1.00
Jingu 21						
*x*_1_	1.00					
*x*_2_	0.98**	1.00				
*x*_3_	0.52	0.35	1.00			
*x*_4_	1.00**	0.99**	0.44	1.00		
*x*_5_	0.96*	0.99**	0.29	0.98**	1.00	
*x*_6_	0.97**	0.99**	0.34	0.98**	1.00**	1.00

*x*_1_ = panicle length; *x*_2_ = panicle diameter; *x*_3_ = panicles ha^−1^; *x*_4_ = grain per panicle; *x*_5_ = 1000-grain weight; *x*_6_ = yield.

* and ** represent significantly difference at *P* = 0.05 and *P* = 0.01, respectively.

### Effect of TBM on protein content in foxtail millet grain

**Fig 3:**
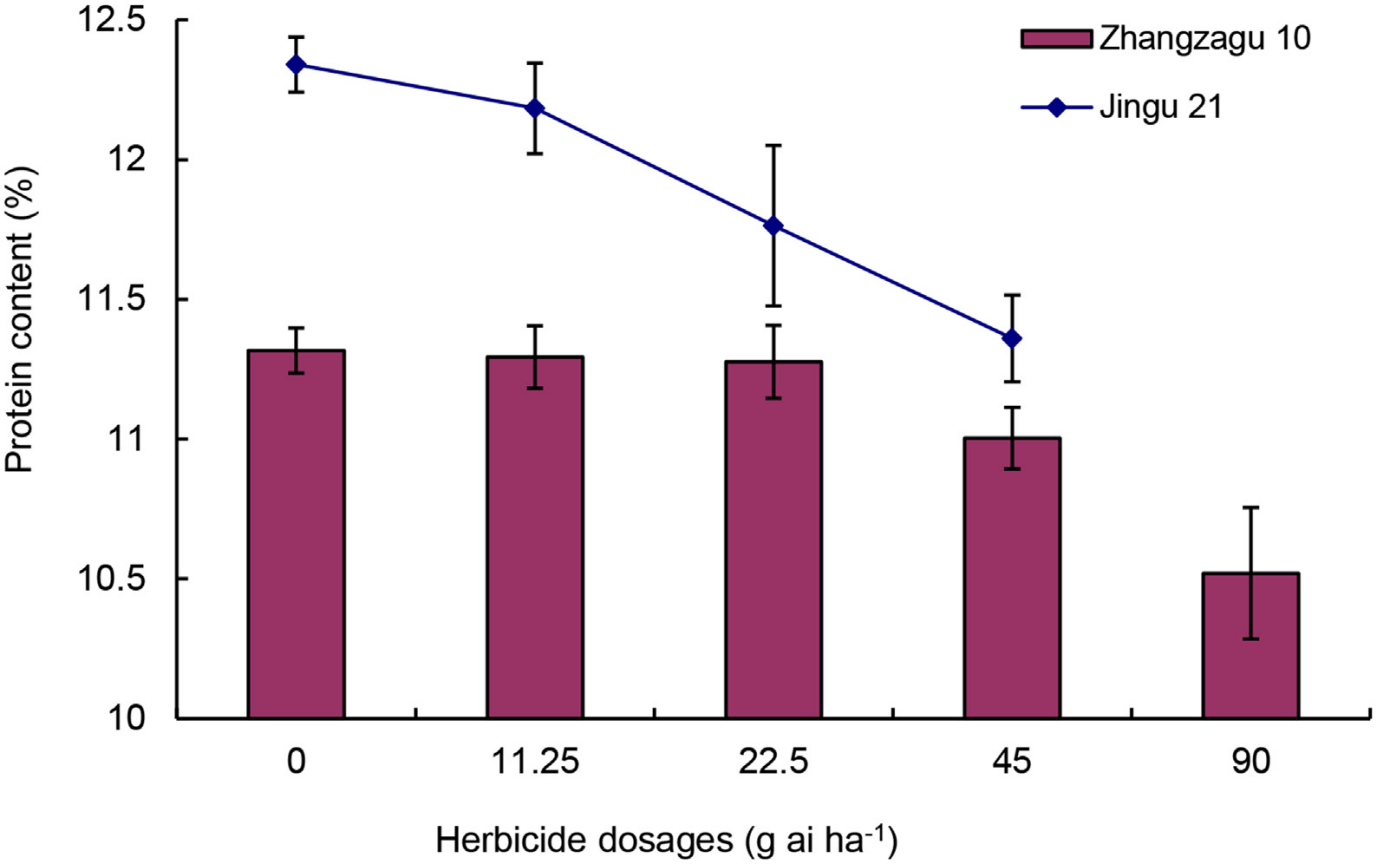
Effect of tribenuron-methyl (TBM) on grain protein content of foxtail millet. Values represent means; vertical bars represent the standard deviation of three separate experiments. The abscissa in the figure represents the dosage of TBM expressed as g ai ha^−1^. Dead Jingu 21 plants were obtained at 90 g ai ha^−1^ TBM.

The grain protein content of foxtail millet decreased with increasing TBM dosages (Fig 3). In Zhangzagu 10, grain protein content significantly decreased at dosages ≥45 g ai ha^−1^ (2.8%), but not at 0–22.5 g ai ha^−1^. In Jingu 21, the grain protein content at 22.5 g ai ha^−1^ (the recommended dosage) was significantly (*P* = 0.05) lower (4.7%) than that of the control.

### Effect of TBM on micromineral concentrations in foxtail millet grain

Grain Zn concentration decreased with increasing dosages of TBM (Table 5). In Zhangzagu 10, grain Zn concentration significantly decreased at 45 g ai ha^−1^, no significant effects were obtained at 11.25 and 22.5 g ai ha^−1^. In Jingu 21, grain Zn concentration was significantly lower at 22.5 g ai ha^−1^. Compared with the control, grain Zn concentration were reduced by 1.4%, 3.3%, 5.8%, and 11.2% in Zhangzagu 10, and by 8.5%, 11.9%, and 14.3% in Jingu 21, respectively.

**Table 5:** Effect of tribenuron-methyl (TBM) on grain mineral concentrations of foxtail millet.

TBM (g ai ha^−1^)	Fe (mg kg^−1^)	Zn (mg kg^−1^)	Mn (mg g^−1^)	Cu (mg kg^−1^)
Zhangzagu 10				
0	33.88±0.77a	36.41±0.55a	11.65±0.10b	5.66±0.18a
11.25	32.15±1.64a	35.91±1.32ab	11.90±0.16a	5.25±0.35a
22.5	31.92±0.93a	35.22±1.50ab	11.94±0.07a	5.19±0.22a
45	28.80±0.90b	34.30±0.37b	11.41±0.14c	4.55±0.26b
90	26.77±0.80c	32.35±0.58c	11.28±0.13c	4.04±0.26c
Jingu 21				
0	36.67±0.31a	37.78±1.79a	11.87±0.15a	5.93±0.35a
11.25	32.65±1.06b	34.58±0.81ab	11.98±0.10a	5.50±0.11b
22.5	30.48±0.52c	33.29±2.77b	11.49±0.21b	5.09±0.23c
45	28.76±1.63c	32.39±0.70b	11.12±0.23c	4.73±0.07c
90	−	−	−	−

Values are expressed as mean ± SD (*n* = 3). Different letters in the same column represent significantly different at *P* = 0.05 level by Duncan’s new multiple range test. “−” represents dead plants.

Grain Mn concentrations of Zhangzagu 10 increased at low dosages (highest Mn concentration obtained at 22.5 g ai ha^−1^) and declined with increasing dosages (lowest Mn concentration obtained at 90 g ai ha^−1^). In Jingu 21, Mn concentrations were slightly higher at 11.25 g ai ha^−1^ relative to that of the control, and decreased with increasing dosage of TBM.

The changes in grain Fe and Cu concentrations of Jingu 21 were similar. At 11.25 g ai ha^−1^ TBM, grain Fe and Cu concentrations were significantly lower than those of the control. However, in Zhangzagu 10, dosage ≥45 g ai ha^−1^ dramatically decreased grain Fe, and Cu concentrations.

## Discussion

The changes in foxtail millet grain yield and quality were remarkable under herbicide TBM stress. The safety of herbicides on crops can be assessed by measuring grain yield, quality, and agronomic traits [28, 29]. In this study, TBM treatment inhibited the growth of foxtail millet, which was reflected by reduced root and shoot biomass (Figs 1 and 2). Additionally, a decline in grain yield of foxtail millet was obtained following TBM treatment (Tables 2 and 3). Wang et al. [17] reported that increasing dosages of TBM negatively impact yield and yield compositions of wheat under artificial dislodgement conditions. In this study, yield reduction varied between years and varieties. Differences in yield reduction might be attributed to varietal properties of herbicide tolerance and climatic conditions over the 2-year study period. The annual average precipitation during the filling stage (August through September) was higher in 2014 versus 2013. Consequently, foxtail millet yield and 1000-grain weight were lower in 2014 than those in 2013.

Foxtail millet yield was determined by grains per panicle, 1000-grain weight, and panicles ha^−1^. Grain yield cannot be determined by a single yield component, because several yield components affect yield to varying degrees at different dosages of TBM. The herbicide directly affected the principal yield components, so as to the yield [28, 30, 31]. Grain yield was positively correlated with grains per panicle and 1000-grain weight, but not with panicles ha^−1^ (Table 4). Panicle length and diameter may represent other good ways of quickly estimating potential yield reduction caused by TBM exposure. A reduction in panicle length and diameter is likely associated with fewer grains per panicle because of a reduced sink. Furthermore, panicle length and diameter were positively correlated with grains per panicle, 1000-grain weight, and yield, (Table 4). Therefore, a reduction in panicle length and diameter would reduce yield components causing a reduction in grain yield. These results were consistent with those obtained by Robinson et al. [28], who reported that several yield components were reduced by decreased agronomic characteristics after exposure to herbicides, leading to reduced yield. In this study, foxtail millet was damaged by high dosages of TBM. As a result, panicle length, panicle diameter, grains per panicle, and 1000-grain weight were significantly reduced, thereby decreasing grain yield. In Zhangzagu 10, the damage was caused by TBM at dosages ≥45 g ai ha^−1^. However, in Jingu 21, the damage was caused at dosages ≥22.5 g ai ha^−1^. Our results revealed that compared with Jingu 21, the hybrid Zhangzagu 10 had higher grain yield and greater herbicide tolerance.

Grain protein content is an important attribute in the nutritional quality of foxtail millet, which contains the highest protein quality among the cereal crops [32]. However, improper application of herbicide TBM affects grain protein content [33]. In this study, the grain protein content of foxtail millet decreased at high dosages of TBM, which may be attributed to the inhibitory action of the herbicide on acetolactate synthase, an enzyme responsible for the biosynthesis of branched chain amino acids (e.g., isoleucine, leucine, and valine) [34]. The significant reduction in grain protein content was observed at dosages ≥45 g ai ha^−1^ in Zhangzagu 10 and at dosages ≥22.5 g ai ha^−1^ in Jingu 21 (Fig 3). This result suggests that the hybrid Zhangzagu 10 has a stronger tolerance to TBM than the conventional Jingu 21. Additionally, TBM at dosages <45 g ai ha^−1^ may not affect protein synthesis in Zhangzagu 10, possibly because of the ability of this variety to metabolize or breakdown the herbicide into non-phytotoxic products [35]. Lum et al. [24] reported that nicosulfuron (a sulfonylurea herbicide) at dosages <105 g ai ha^−1^ caused little inhibition of protein synthesis in maize.

Grain mineral content is affected by a number of factors, such as varietal properties, soil mineral levels, efficiency of mineral uptake, and weed control methods [36, 37]. In China, Jingu 21 is a high-quality foxtail millet variety. Compared with Jingu 21, Zhangzagu 10 has higher grain yield but lower quality [38]. In this study, grain mineral concentrations were consistently higher in Jingu 21 than in Zhangzagu 10 without TBM treatment, reflecting the varietal properties. As shown in Table 1, there were significant differences among the soil mineral levels, which were all within the normal range. All treatments were conducted under similar soil conditions each year, therefore, the variances in the results were attributed to the herbicide. Treatment with TBM ≤22.5 g ai ha^−1^ did not affect grain Fe, Mn, Cu, and Zn contents in Zhangzagu 10. However, grain mineral contents in Jingu 21 significantly decreased at dosages ≥11.25 g ai ha^−1^. Gugala et al. [37, 39] reported that herbicides affect grain mineral concentrations. Zn, Mn, Fe, and Cu concentrations in foxtail millet grain decreased in response to increased TBM dosages, probably due to reduced root activity [40]. The herbicide tolerance of hybrid Zhangzagu 10 probably helped to protect the root against damage at dosages ≤22.5 g ai ha^−1^. Future studies should investigate the effect of TBM on more genotypes of foxtail millet, and the molecular and biochemical mechanisms responsible for TBM tolerance.

## Conclusion

The recommended dosage of TBM (22.5 g ai ha^−1^) was not safe for the conventional variety Jingu 21, but relatively safe for the hybrid variety Zhangzagu 10. At this dosage, the herbicide significantly decreased grain yield and biomass, grain protein content, and grain Mn, Cu, Fe, and Zn concentrations in Jingu 21, but not in Zhangzagu 10. The hybrid variety Zhangzagu 10 showed a stronger tolerance to TBM than the conventional variety Jingu 21.

## Supporting Information

S1 Table The raw data. The table matched to Fig 1.
(XLS)

S2 Table The raw data. The table matched to Fig 2.
(XLS)

S3 Table The raw data. The table matched to Table 2.
(XLS)

S4 Table The raw data. The table matched to Table 3.
(XLS)

S5 Table The raw data. The table matched to Table 4.
(XLS)

S6 Table The raw data. The table matched to Fig 3.
(XLS)

S7 Table The raw data. The table matched to Table 5.
(XLS)
